# A New ELISA to Overcome the Pitfalls in Quantification of Recombinant Human Monoclonal Anti-HBs, GC1102, by Commercial Immunoassays

**DOI:** 10.1186/s12575-018-0083-8

**Published:** 2018-09-27

**Authors:** Yong Won Shin, Dong-Hyung Cho, Gi Won Song, Se-Ho Kim

**Affiliations:** 1 R&D center, GC Pharma, Yongin, 16924 Korea; 2 School of Life Science, Kyungpook National University, Daegu, 41566 Korea; 3 Division of Hepatobiliary Surgery and Liver Transplantation, Department of Surgery, University of Ulsan College of Medicine and Seoul Asan Medical Center, Seoul, 05505 Korea; 4 Division of Biomedical Convergence, College of Biomedical Science, Kangwon National University, Chuncheon, 24641 Korea; 5 Institute of Bioscience and Biotechnology, Kangwon National University, Chuncheon, 24341 Korea

**Keywords:** Anti-HBs, ELISA, GC1102, Immunoassay, Indirect ELISA, Quantification, Sandwich ELISA

## Abstract

Several methods for the quantification of human anti-HBs, an antibody to hepatitis B surface antigen (HBsAg), have been developed based on enzyme reaction, chemiluminescence, fluorescence, and radioactivity for application to human serum or plasma. Commercial anti-HBs immunoassay kits use a sandwich method in which a bridge is formed by the anti-HBs between a HBsAg immobilized solid matrix and the labeled HBsAg. However, this direct sandwich enzyme-linked immunosorbent assay (ELISA) is insufficient to accurately evaluate the activity of the human monoclonal anti-HBs, GC1102. As an alternative, we developed an indirect anti-HBs ELISA (anti-HBs qELISA_v.1) that improved detection of anti-HBs. In this current study, we further optimized this indirect method to minimize nonspecific binding of human serum, by employing incubation buffers containing animal serum, Tween 20, skim milk, and a low pH washing buffer. This new and improved method, termed anti-HBs qELISA_v.2, showed accurate quantification of plasma-derived hepatitis B immune globulin (HBIG) and was comparable to results obtained with commercial ELISA (*r* = 0.93) and RIA (*r* = 0.85) kits. Further, the GC1102 in human serum could be precisely measured using the anti-HBs qELISA_v.2 without limitations of nonspecific binding.

## Background

Hepatitis B is caused by infection of the liver by the hepatitis B virus (HBV). According to the World Health Organization (WHO) report in 2015, approximately 257 million people were infected by HBV and 887,000 people succumbed to HBV-induced death, mostly from liver cirrhosis and hepatocellular carcinoma [1, 2].

Hepatitis B immune globulin (HBIG), prepared from hepatitis B vaccine-boosted plasma, is being used effectively for HBV prophylaxis and for preventing hepatitis B recurrence after liver transplantation in patients with hepatitis B-induced liver complications [3–6]. However, production of HBIG has several limitations including limited plasma supply, low specific activity, and the potential for infectious contaminants from human plasma [7, 8]. To overcome these limitations, a recombinant human monoclonal anti-HBs, GC1102 (formerly known as HB-C7A), has been developed [8, 9]. It is generally accepted that a sustained level of at least 10 IU/L of anti-HBs antibody is protective against HBV infection. For liver transplant recipients, quantitative measurement of anti-HBs is used in the management of HBIG prophylaxis, to maintain anti-HBs levels of at least 100 or 200 IU/liter, according to different guidelines. All these recommendations imply that the measurement of anti-HBs levels by different assays is accurate and consistent, yielding comparable quantitative results in various laboratories and countries [10] and references therein.

Generally, quantification of anti-HBs is carried out using a direct sandwich type of enzyme-linked immunosorbent assay (ELISA), which relies on a HBsAg-coated solid matrix and horseradish peroxidase (HRP) labeled-HBsAg for the detection and estimation of anti-HBs [11]. Several modifications of this standard method exist, wherein different solid matrix and detection compounds are used [12]. However, the direct sandwich method does not accurately evaluate the quantity of GC1102, due to the inherent limitations of the method. To solve this limitation and improve the detection of anti-HBs, we devised an indirect method of ELISA [8]. This indirect method uses a similar HBsAg-immobilized microplate as sandwich ELISA, however it makes use of goat anti-human IgG to detect bound anti-HBs, instead of HBsAg. This anti-HBs qELISA_v.1 showed accurate quantification of recombinant anti-HBs in cell culture media, PBS buffer and monkey serum, but failed to quantify precise levels of anti-HBs in human serum due to a high nonspecific binding. Therefore, we further optimized the method to develop anti-HBs qELISA_v.2, which shows reduced nonspecific binding, and was comparable to commercial kits for measurements of anti-HBs levels in human serum.

## Methods

### Establishment of Indirect ELISA Method (Anti-HBs qELISA_v.1)

The indirect ELISA method for measuring anti-HBs quantity was previously described [8]. Briefly, anti-HBs in the sample, bound to the HBsAg-immobilized microplate, were detected by a goat anti-human IgG (Fab specific)-peroxidase conjugate. PBS-T (phosphate-buffered saline, pH 7.2, containing 0.05% Tween 20) and 1% BSA-PBS were used as washing and sample dilution buffer, respectively.

### Anti-HBs Reference for Activity Measurement

The National Biological Reference Standard Human Hepatitis B Immunoglobulin (reference standard, 95.45 IU/vial, Code No. 08/026) was obtained from The Korea Ministry of Food and Drug Safety and reconstituted by following the instructions provided.

### Comparison of ELISA Titer between HBIG and GC1102 Using Anti-HBs qELISA_v.1

The plasma-derived HBIG that we used for comparison was Hepabig® (GC pharma, Yongin, Korea), and it was estimated to be 1 IU/mg. The Hepabig® and GC1102 were serially diluted and ELISA was performed as previously described [8].

### Activity Measurement of GC1102 with Commercial Kits

ARCHITECT anti-HBs (Abbott Ireland Diagnostics Division, Sligo, Ireland) was used as a standard assay, which is a chemiluminescent microparticle immunoassay (CMIA), for the quantitative determination of anti-HBs in human serum and plasma.

### Optimization of Indirect ELISA for Reducing Non-specific Binding (Anti-HBs qELISA_v.2)

The HBsAg (LG Chemical, Seoul, Korea) was immobilized to the wells of microplate (Nunc Immuno Module, Maxisorp; NUNC, Roskilde, Denmark), as previously described [8]. The reference standard and serum samples were diluted in a sample dilution buffer which consisted of PBS, pH 7.2 (Lonza, Allendale, NJ, USA) containing 0.1% skim milk (BD Bioscience, Franklin Lakes, NJ, USA), 10% bovine serum (Thermo Fisher, Waltham, MA, USA), and 0.05% Tween 20 (Sigma-Aldrich, St. Louis, MO, USA). Then, 100 μL of diluted standard or serum was added to the HBsAg-coated microplate and incubated for 60 min at room temperature (RT). Plates were washed 5 times with washing buffer composed of 20 mM sodium acetate (Sigma-Aldrich) buffer, pH 4.0, containing 150 mM sodium chloride (Sigma-Aldrich) and 0.05% Tween 20. Then, 100 μL of goat anti-human IgG (Fc specific)-peroxidase conjugate (Sigma-Aldrich), which was diluted to 20,000-fold in secondary antibody dilution buffer composed of PBS (pH 7.2) containing 10% goat serum (Thermo Fisher), was added to the microplate and incubated for 30 min at RT. After washing 5 times with washing buffer, 100 μL of TMB substrate solution (KPL, Gaithersburg, MD, USA) was added and samples were incubated for 30 min at RT. The enzyme reaction was stopped by addition of 100 μL of 1 N sulfuric acid (Sigma-Aldrich) and the absorbance was measured at 450 nm. Anti-HBs reference standards consisting of 0, 10, 50, 150, 500, and 1000 IU/L were employed, and the standard curve was fitted by 4-parameter logistic regression.

### Preparation of Human Serum Samples

Human serum samples were prepared from 9 healthy volunteers who participated in phase 1 clinical study at Seoul Asan Medical Center for a safety evaluation of GC1102. Control group was administered 10,000 IU of I.V.-Hepabig® (GC Pharma, Yongin, Korea). Blood was collected at 19 different time points for 12 weeks after a single administration. Another 19 serum samples that were HBsAg (+) and anti-HBs (−) were obtained from the Seoul Asan Medical Center to evaluate the assay method for reduction of nonspecific binding.

Blood collection and its analyses were approved by the Institutional Review Board (IRB) of Asan Medical Center (Seoul, Korea; IRB No. 2008–0129).

### Comparison of Anti-HBs qELISA_v.2 with Commercial Kits

The anti-HBs qELISA_v.2 was compared with two commercially available anti-HBs kits, HBsAb-RIA (Beijing North Institute of Biological Technology, Beijing, China) and GENEDIA Anti-HBs ELISA 3.0 (GC Medical Sciences, Yongin, Korea), for human serum samples. Assays were performed according to the manufacturers’ instructions. The correlation coefficients for linear regressions (*R*^2^) for the relationships between the anti-HBs qELISA_v.2 and the two commercial methods were evaluated using the Excel 2010 program (Microsoft, Seattle, WA, USA).

## Results and Discussion

### Discrepancy of Expected Value of GC1102 Estimation between Anti-HBs qELISA_v.1 and Commercial ELISAs

**Fig. 1:**
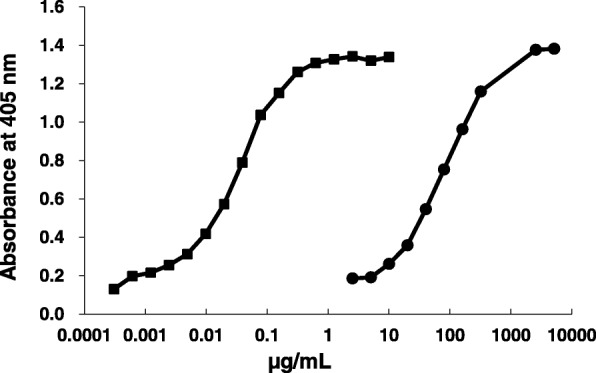
Comparison of titer of GC1102 (■) and Hepabig® (●) by indirect anti-HBs qELISA_v.1. The Hepabig® used was 1.3 IU/mg

GC1102 is a recombinant human monoclonal anti-HBs IgG1 derived from Fab clone HB4–9, which was selected from a phage library [13]. Establishment of GC1102-producing Chinese Hamster Ovary (CHO) cells, and characterization of GC1102 activity, affinity, and specificity was previously reported [8]. The activity of GC1102 is approximately 2600 IU/mg and that of Hepabig® is approximately 1.3 IU/mg, based on the WHO International Unit of anti-hepatitis B immunoglobulin [8]. When the protein quantities of GC1102 and Hepabig® (1.3 IU/mg) were compared, GC1102 exhibited 2300 times higher activity, which is approximately 3000 IU/mg (Fig. 1). This activity of GC1102 was manifested in hydrodynamic mouse model experiments in which 50 IU of GC1102 (estimated by the assumption that the activity of GC1102 was 3000 IU/mg) exhibited similar HBV neutralizing activity as 50 IU of Hepabig® (data not shown).

**Fig. 2:**
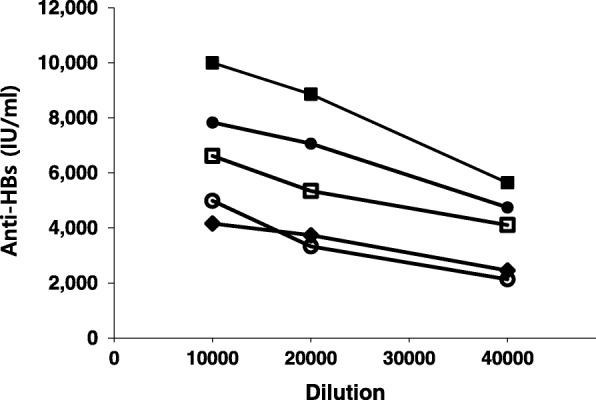
Activity measurement of GC1102 by Abbott ARCHITECT Anti-HBs against different dilution solutions for 10,000-, 20,000-, and 40,000-fold dilutions. Dilution solutions were 1% BSA in PBS (■), 5% BSA in PBS (●), 10% BSA in PBS (**□**), 20% BSA in PBS (♦), and dilution buffer in the kit (**○**)

A commercial kit, ARCHITECT Anti-HBs, was adopted for quality control (QC), nonclinical, and clinical studies for GC1102. ARCHITECT Anti-HBs was examined; however, this did not exhibit the expected activity of GC1102 and there was no dilution linearity observed for the diluent provided in the kit and several concentrations of bovine serum albumin (BSA) solutions in PBS (pH 7.2) (Fig. 2). For a 10,000-fold dilution of GC1102, this kit exhibited measured values ranging between 4000 and 10,000 IU/ml for different dilution buffers used. Similar variations of activity were observed in 20,000- and 40,000-fold dilutions in different dilution buffers. The dilution buffer provided in the kit exhibited 5000, 3500, and 2200 IU/ml against 10,000, 20,000, and 40,000-fold dilutions (Fig. 2). Similar nonlinearities were observed for different dilution buffers.

Further, when the activity of GC1102 was measured by GENEDIA Anti-HBs ELISA 3.0, it gave a value of 624.9 ± 89.5 IU/mg. Other commercial ELISA kits such as ETI-AB-AUK-3 (anti-HBs) (DiaSorin S.p.A., Saluggia, Italy), Enzygnost Anti-HBs ELISA III (Dade Behring, Marburg, Germany) and Monolisa Anti-HBs PLUS (Bio-Rad, Marnes-la-Coquette, France) exhibited similar trend as that of GENEDIA Anti-HBs ELISA 3.0.

**Fig. 3:**
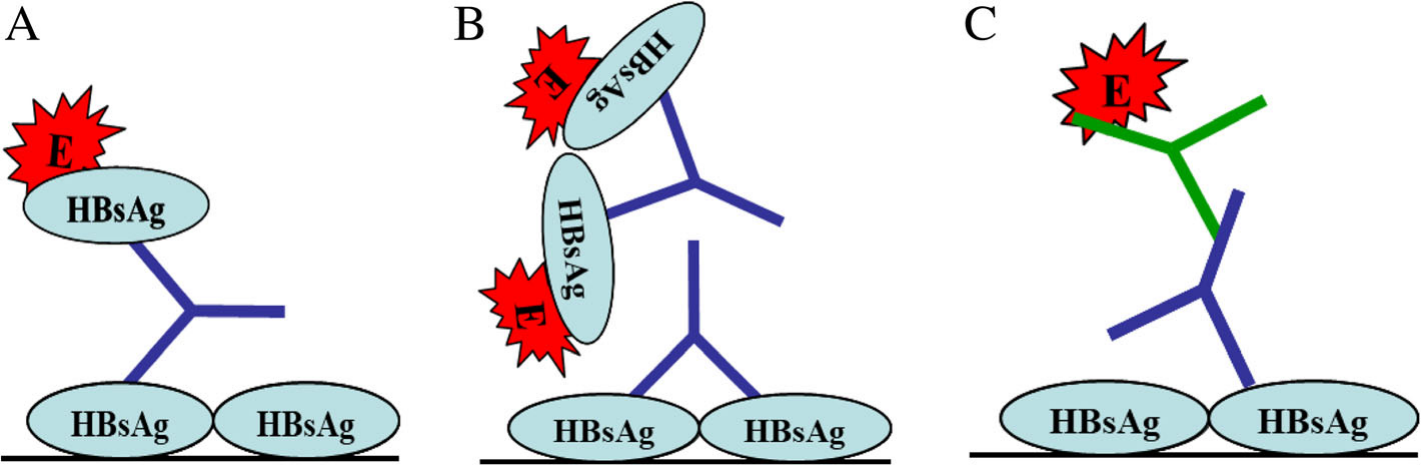
Schematic illustration for measuring anti-HBs titers by ELISA. Blue colored Y denotes GC1102 and green colored Y denotes goat anti-human IgG. “E” denotes an enzyme in this ELISA. **a** General principle of sandwich ELISA for measuring anti-HBs. Anti-HBs in the sample forms a bridge between the immobilized HBsAg and the labeled HBsAg, and signals produced thereby are measured. **b** Possibility of not forming the bridge due to a masking of antibody binding sites by immobilized and/or labeled HBsAg. **c** Indirect ELISA for correct quantification of anti-HBs. Any anti-HBs bound to the immobilized HBsAg can be detected by HRP labeled goat anti-human IgG

One reason for such low estimation of GC1102 activity by commercial kits can be as explained below and depicted in Fig. 3a and b. The sandwich ELISA works on the principle that immobilized HBsAg and labeled HBsAg are bridged by anti-HBs, and signals produced thereby, by the labeled enzyme, is measured (Fig. 3a). However as shown in Fig. 3b, there is a possibility that the bridge is not formed due to masking of antibody binding sites by immobilized and/or labeled HBsAg. If immobilized or labeled HBsAg occupied the two binding sites of GC1102, there would be no bridge formation, resulting in loss of binding activity of GC1102.

In contrast, using the new method, which does not rely on bridge formation, there is no possibility of not detecting any HBsAg-bound GC1102 (Fig. 3c). As indicated by our results, any anti-HBs bound to the immobilized HBsAg could be detected by HRP labeled goat anti-human IgG. Furthermore, the activity of GC1102 measured by this new method reflected the expected activity of GC1102, predetermined by comparing with Hepabig®. Accordingly, this new indirect method was adopted for QC, nonclinical, and clinical studies.

### Optimization of Anti-HBs qELISA_v.1 for Detection in Human Serum Samples

Despite the usefulness of anti-HBs qELISA_v.1 in QC and nonclinical studies, it exhibited high nonspecific binding for human serum samples obtained from phase I clinical studies of GC1102. To resolve this problem of nonspecific binding, the sample dilution buffer for human serum was changed from 1% BSA-PBS to PBS, pH 7.2, containing 0.1% skim milk, 10% bovine serum and 0.05% Tween 20. Bovine serum could be considered the most effective reagent to reduce the non-specific binding of endogenous human IgG. Tween 20 is often used to prevent non-specific interactions between a solid surface and proteins and to block the empty space of the microwell plate [14]. Skim milk is widely used as blocking reagents for ELISA [15]. Additionally, Fujii et al. reported that normal rabbit serum could minimize the non-specific binding of IgG onto the surface of a microtiter plate [16].

**Fig. 4:**
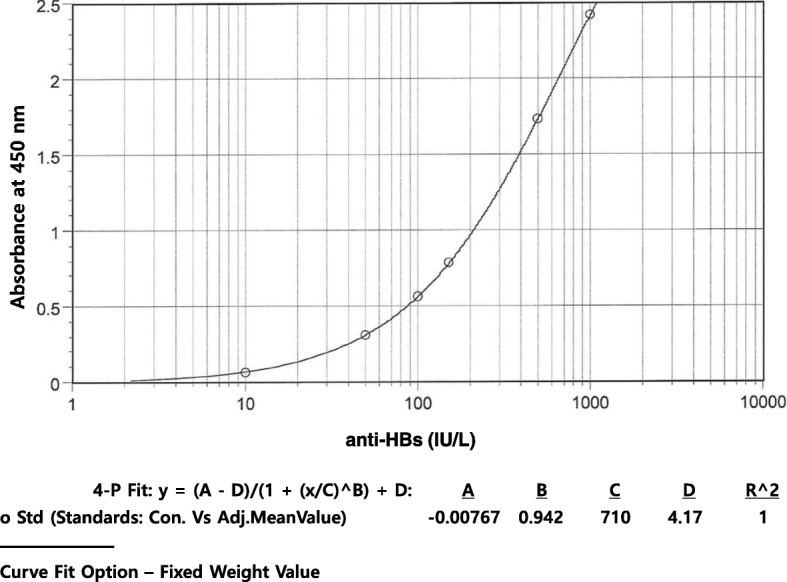
Representative standard curve of anti-HBs qELISA_v.2 fitted by 4-parameter logistic regression. Anti-HBs reference standards consisted of 0, 10, 50, 150, 500, and 1000 IU/L. The duplicate readings for each standard were averaged and the average zero standard optical density was subtracted. In equation y = (A-D)/(1 + x/C)^∧^B) + D, A = − 0.00767, B = 0.942, C = 710, and D = 4.17 and R^∧^2 = 1

Next, the secondary antibody dilution buffer was changed from 1% BSA in PBS to 10% goat serum in PBS. Goat serum was employed to reduce the non-specific binding of goat anti-human antibodies. Further the washing buffer was changed from PBS-T to 20 mM acetate buffer, pH 4.0, containing 150 mM sodium chloride and 0.05% Tween 20. The assay conditions of anti-HBs qELISA_v.1 and anti-HBs qELISA_v.2 are summarized in Table 1, and the representative standard curve is shown in Fig. 4.

**Table 1:** Comparison of the features of anti-HBs qELISA_v.1 and anti-HBs qELISA_v.2

	Anti-HBs qELISA_v.1	Anti-HBs qELISA_v.2
Sample dilution buffer	1% BSA in PBS, pH 7.2	10% BSA, 0.1% skim milk and 0.05% Tween 20 in PBS, pH 7.2
Secondary antibody dilution buffer	1% BSA in PBS, pH 7.2	10% Goat Serum in PBS, pH 7.2
Washing buffer	PBS-T (PBS, pH 7.2, containing 0.05% Tween 20)	20 mM sodium acetate buffer, pH 4.0, containing 150 mM sodium chloride and 0.05% Tween 20

The anti-HBs qELISA_v.2, together with the anti-HBs qELISA_v.1, were used to evaluate nonspecific binding in 19 HBsAg (+) and anti-HBs (−) samples provided from Seoul Asan Medical Center. The anti-HBs qELISA_v.2 did not exhibit nonspecific binding. In contrast, the anti-HBs qELISA_v.1 exhibited high nonspecific binding, resulting in false anti-HBs titers from 478 to 2700 IU/L (Table 2).

**Table 2:** Summary of analyses of 19 HBsAg(+) and anti-HBs(−) samples from hepatitis B patients

No.	HBsAg	Anti-HBs Activity (IU/L)	
		Anti-HBs qELISA_v.1	Anti-HBs qELISA_v.2
1	(+)	1014	< 100
2	(+)	1023	< 100
3	(+)	970	< 100
4	(+)	1189	< 100
5	(+)	1691	< 100
6	(+)	1734	< 100
7	(+)	1757	< 100
8	(+)	1662	< 100
9	(+)	967	< 100
10	(+)	478	< 100
11	(+)	974	< 100
12	(+)	1770	< 100
13	(+)	2341	< 100
14	(+)	2701	< 100
15	(+)	2644	< 100
16	(+)	1228	< 100
17	(+)	1266	< 100
18	(+)	1358	< 100
19	(+)	1305	< 100

There have been no reports of employing a low pH-washing buffer in immunoassay. However, incorporation of a low pH wash with a buffer having pH 5 was effective in removing non-specifically adsorbed phages during cell-based biopanning [17]. Additionally, the incubation of serum samples at acidic pH (pH 3.5) during ELISA, for the quantitation of IL-13 in human serum, reduced nonspecific binding [18].

### Correlation of Anti-HBs qELISA_v.2 with Commercial Kits

**Fig. 5:**
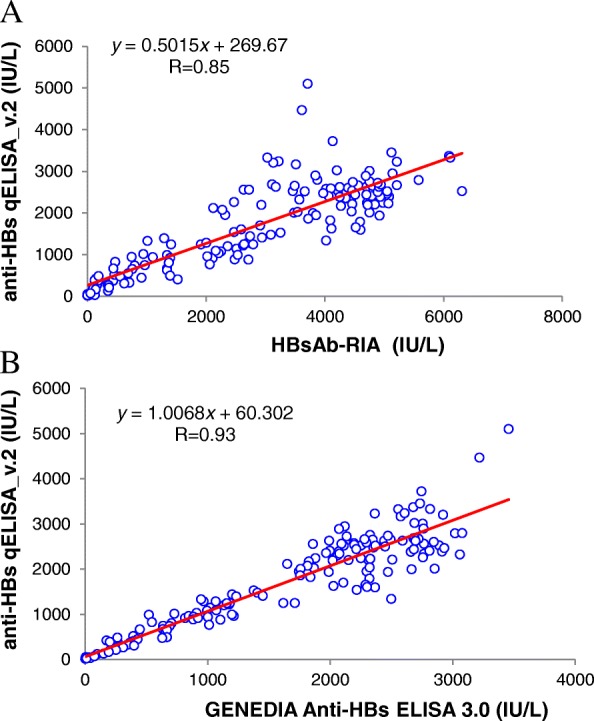
Overall correlation observed during anti-HBs measurement from 173 samples by **a** HBsAb-RIA vs. anti-HBs qELISA_v.2 and **b** GENEDIA Anti-HBs ELISA 3.0 vs. anti-HBs qELISA_v.2. The line corresponds to the best fit correlation and its equation is presented

Human serum samples were prepared from 9 healthy volunteers who participated in phase 1 clinical study at Seoul Asan Medical Center for a safety evaluation of GC1102. They were administered 10,000 IU of I.V.-Hepabig® as a control group. Because conventional immunoassay kits exhibited lower activity of GC1102, I.V.-Hepabig® administered serum samples were employed for comparison. Blood was collected at 19 different time points for 12 weeks after a single administration. All 173 serum samples from volunteers were evaluated by three different anti-HBs measuring methods, i.e., HBsAb-RIA, GENEDIA Anti-HBs ELISA 3.0, and anti-HBs qELISA_v.2 and correlations between each method were compared. Comparison of the methods by functional relationship statistics showed that the regression coefficient *r*, for HBsAb-RIA vs. anti-HBs qELISA_v.2 was 0.85 (Fig. 5a), and that for Anti-HBs ELISA 3.0 vs. anti-HBs qELISA_v.2 was 0.93 (Fig. 5b).

The comparison between DiaSorin LIAISON® anti-HBs II and Abbott Architect anti-HBs tests were reported to have a strong correlation (*r* = 0.92) [19]. However, both kits used the same principle of chemiluminescence immunoassay, with differences in the source of HBsAg and incubation step. The correlation between the quantity of serum anti-HBs levels measured by Modular E170 and Architect i2000 was also high (*r* = 0.92) [20]. When four different methods of Abbott AxSYM AUSAB, Enzygost Anti-HBs II, Roche Cobas Core Anti-Hbs Quant EIA II and Elecsys Anti-HBs were compared, the correlation coefficient ranged between 0.62 ~ 0.87 [21]. Considering the correlations reported above, the novel anti-HBs qELISA_v.2 system that we established seems to be comparable to the commercially recognized anti-HBs quantification systems. Moreover, the anti-HBs qELISA_v.2 could be successfully used to evaluate the serum samples from phase 1 clinical study of GC1102 (data not shown).

In conclusion, we have established a new anti-HBs quantification ELISA by minimizing or preventing nonspecific binding of human serum samples. This method can be used for the determination of GC1102 for QC, nonclinical, and clinical samples analyses, which is not possible by the current commercial anti-HBs quantitation kits that are based on the sandwich immunoassay method.

## Abbreviations

Anti-HBs: Antibody to hepatitis B virus surface antigen
BSA: Bovine serum albumin
CMIA: Chemiluminescent magnetic microparticle immunoassay
ELISA: Enzyme-linked immunosorbent assay
HBIG: Hepatitis B immune globulin
HBsAg: Hepatitis B virus surface antigen
HBV: Hepatitis B virus
IgG: Immunoglobulin G
PBS: Phosphate-buffered saline
RIA: Radioimmunoassay
